# Predictors of Depression and Death Anxiety Among Filipino Older Adults: The Roles of Meaning in Life, Self-Esteem, and Life Satisfaction

**DOI:** 10.3390/ijerph23050654

**Published:** 2026-05-14

**Authors:** Gil P. Soriano, Mark Edllin R. Rafol, Ezekiel Zachary C. Samonte, Reena A. Asturias, Carissa Juliana R. Balaria, Mars Ian A. Silud, Feni Betriana, Kathyrine A. Calong Calong

**Affiliations:** 1 Department of Nursing, College of Allied Health, National University, Manila 1008, Metro Manila, Philippines; 2 Nursing and Patient Care Services Division, Makati Medical Center, Makati 1229, Metro Manila, Philippines; 3 Nursing Services Division, Pasay City General Hospital, Pasay City 1300, Metro Manila, Philippines; 4 College of Nursing, Kolehiyo ng Lungsod ng Dasmariñas, Dasmariñas City 4114, Cavite, Philippines; 5 College of Nursing, Wesleyan University-Philippines, Cabanatuan City 3100, Nueva Ecija, Philippines; 6 College of Nursing, Cristal e-College, Panglao 6340, Bohol, Philippines; 7 Center for Biomedical Research, National Research and Innovation Agency (BRIN), Cibinong 16911, Indonesia; feni004@brin.go.id; 8 College of Nursing, San Beda University, Manila 1005, Metro Manila, Philippines; kathyrine3@gmail.com

**Keywords:** death anxiety, depression, life satisfaction, meaning in life, self-esteem

## Abstract

**Highlights:**

**Public Health Relevance—How does this work relate to a public health issue?**

- Depression and death anxiety are prevalent yet underrecognized mental health concerns among older adults, particularly in community settings in low- and middle-income countries such as the Philippines.
- This study situates these outcomes within broader public health determinants by examining psychosocial and existential factors (e.g., meaning in life, self-esteem, and life satisfaction) that influence mental health in aging populations.

**Public Health Significance—Why is this work of significance to public health?**

- The findings highlight that psychological and existential factors are significant correlates of mental health outcomes, underscoring the need to integrate mental health and psychosocial care into healthy aging frameworks.
- By focusing on Filipino older adults, this study addresses a critical gap in culturally contextualized public health evidence, particularly in collectivist societies where family, spirituality, and social roles shape well-being.

**Public Health Implications—What are the key implications or messages for practitioners, policy makers, and/or researchers?**

- Community-based health programs should incorporate routine screening for depression and death anxiety alongside interventions that promote meaning in life and life satisfaction, such as life review, social engagement, and spiritual care initiatives.
- Policymakers and researchers should prioritize culturally responsive mental health strategies and longitudinal research to better understand and address the complex interplay between existential well-being and mental health in aging populations.

**Abstract:**

In sociocultural situations where aging is firmly associated with family roles, spirituality, and reliance, depression and death anxiety are prevalent mental health issues among older individuals. Although their functions may vary depending on the situation, psychological resources, such as purpose in life, self-worth, and life satisfaction, are often considered protective in later life. This descriptive–correlational study examined the relationships between meaning in life, self-esteem, life satisfaction, depression, and death anxiety among 119 community-dwelling older Filipinos aged 60 years and older. Data were analyzed using descriptive statistics, Pearson correlation, and multiple linear regression in JAMOVI version 2.7.6. The standardized instruments included the Meaning in Life Questionnaire, Rosenberg Self-Esteem Scale, Satisfaction with Life Scale, Revised Death Anxiety Scale, and Beck Depression Inventory. Regression analysis revealed that while meaning in life and life satisfaction independently predicted higher death anxiety, they independently predicted lower depression. After adjustment, self-esteem predicted neither outcome. These results suggest that psychological resources have distinct effects on mental health in later life, acting as protective factors against depressive symptoms and raising awareness of mortality. To promote healthy aging among older individuals, culturally responsive therapies that address existential factors and emotional well-being are necessary.

## 1. Introduction

Although death is a universal human experience, the realization of mortality causes significant psychological reactions in people of every background. When older individuals deal with physical decline, chronic sickness, and loss of significant others, death becomes more prominent to them [1,2]. A fear or worry related to death and dying, death anxiety, can be a conscious or unconscious psychological condition brought on by the realization of one’s own mortality [3]. Death anxiety has been widely examined across both clinical and non-clinical populations, with variations influenced by psychological and contextual factors [4]. High levels of death anxiety in older individuals have been linked to decreased psychological well-being, increased depressive symptoms, and diminished life satisfaction [5]. Chronic disease, social isolation, and post-pandemic uncertainty intensify death anxiety and compromise mental health in aging populations [6].

Depression and death anxiety are significant global mental health concerns among older adults, and their prevalence is increasing alongside population aging. Depression affects approximately 7% of the global older population and is a leading contributor to disability and reduced quality of life [7]. Death anxiety, although less frequently quantified, has been recognized as a transdiagnostic construct that influences multiple psychological conditions [2].

Depression is another common mental health issue found in later age, which has a major impact on mortality, functional ability, and quality of life [8,9]. Depression should not be viewed as an inevitable consequence of aging, even though it incorporates biological and psychological changes [9]. The symptoms of depression commonly reflect a combination of physical illnesses, existential issues, and loss [10]. Meaning in life, depression, anxiety, and loneliness are dynamically correlated among older primary care patients, with higher meaning in life being associated with fewer symptoms of depression and anxiety [5].

The concept of meaning in life (MIL) refers to a person’s sense of direction, consistency, and significance in existence [11]. A strong sense of purpose in life tends to make older individuals more resilient to loss and emotional pain [12]. Meaning in life is a psychological resource that mediates the connection between well-being and mortality awareness. Self-esteem mediates the relationship between death anxiety and meaning in life [5]. Depression and death anxiety inversely correlate with psychological well-being and purpose in life, with loneliness serving as a partial mediating factor [13].

From an existential perspective, mortality awareness is a fundamental human condition that shapes meaning-making processes and psychological adjustment [14]. Lifespan developmental theories further suggest that successful aging involves adaptation to loss, redefinition of purpose, and maintenance of psychological coherence despite physical decline.

Another important factor influencing emotional responses to mortality is self-esteem, a stable sense of one’s own worth and ability. Self-esteem serves as a protective factor against death anxiety by validating one’s cultural and symbolic value [15]. Individuals with positive self-esteem experience lower depression rates and a diminished emotional response to mortality salience [16,17]. However, cultural differences affect this relationship. Family and spirituality may provide existential security in collectivist countries, such as the Philippines, more so than individual self-concept [18]. Relational and spiritual dimensions often shape emotional experiences in collectivist contexts, rather than individual appraisal alone [19]. This is particularly relevant in the Filipino setting, where family interconnectedness and religiosity may influence how older adults interpret psychological distress and mortality [18].

Lastly, life satisfaction, the evaluative component of subjective well-being, reflects people’s cognitive assessment of the quality of their lives [20]. Psychological distress and existential fear are associated with poor life satisfaction, whereas emotional stability is associated with high life satisfaction [21,22]. Additionally, individuals with higher levels of life satisfaction, particularly those with close family relationships, were associated with lower levels of depression and better adaptation to aging [23]. Conversely, higher levels of death anxiety have been associated with lower life satisfaction, particularly when loneliness and declining health status are combined [9,24].

In the Philippines, life expectancy has steadily increased to approximately 71 years, contributing to a growing population of older adults with evolving psychosocial needs [6]. Despite this demographic shift, empirical research examining existential and psychological outcomes such as death anxiety and depression among Filipino older adults remains limited, highlighting a critical gap in the literature.

Despite these realizations, little research has examined how life satisfaction, self-esteem, and meaning in life relate to depression and death anxiety in older Filipino adults. Thus, the purpose of this study was to determine how death anxiety and depression among older Filipino adults relate to life satisfaction, self-esteem, and meaning in life.

## 2. Materials and Methods

### 2.1. Research Design and Sampling Technique

The study is descriptive-correlational research to determine the relationship of meaning in life, self-esteem, and life satisfaction with death anxiety and depression among older adults. Convenience sampling was used to select participants for the study.

Convenience sampling was employed due to logistical constraints and the need to access community-dwelling older adults through existing barangay networks, a common approach in community-based research where probabilistic sampling is not feasible [25].

### 2.2. Setting of the Study

This study was conducted in a selected city in Metro Manila with 201 barangays divided into two districts. The researchers collaborated with the Office of the Senior Citizen Affairs (OSCA) and the captains of different barangays. The target population consisted of older adults living within the selected city. Respondents must meet the following inclusion criteria: (1) male or female, aged 60 years old or above; (2) living in the selected city; (3) willing to participate in the study; and (4) knows how to read and write.

Older adults with severe chronic illness, significant functional impairment, or those residing in institutional care facilities were not included in the study, as the ability to complete self-administered questionnaires was required for participation. While demographic data included general indicators such as age and income, specific clinical variables (e.g., comorbidities and disability status) were not systematically measured.

### 2.3. Research Instrument

The researcher would utilize a standardized research instrument, which includes a demographic profile sheet, the Meaning in Life Questionnaire, the Rosenberg Self-Esteem Scale, the Revised Death Anxiety Scale, and the Beck Depression Inventory. Reliability estimates reported in this study refer to internal consistency, as measured using Cronbach’s alpha coefficients.

#### 2.3.1. Demographic Profile Sheet

This section contains data on the participants, including age in years, gender, civil status, religion, education, and income.

#### 2.3.2. Meaning in Life Questionnaire (MLQ)

This was developed by Steger et al. [11], which assessed two dimensions of meaning in life using 10 items: (1) Presence of Meaning—(Items 1, 4, 5, 6, and 9) and (2) Search for Meaning—(Items 2, 3, 7, 8, and 10). Using a 7-point Likert scale, item responses ranged from absolutely untrue to absolutely true. It demonstrated acceptable internal consistency, with Cronbach’s alphas of 0.74 for presence and 0.81 for search [4]. In this study, the reliability coefficient was 0.905.

#### 2.3.3. Rosenberg Self-Esteem Scale (RES)

This scale was developed by Rosenberg [26] and was used to measure self-esteem among older adults. It is a 10-item self-report measure of global self-esteem. It consists of 10 statements related to the overall feelings of self-worth or self-acceptance. RES demonstrated both a unidimensional and a two-factor structure to the scale: self-confidence (Items 1, 3, 4, 7, and 10) and self-depreciation (Items 2, 5, 6, 8, and 9). All items are answered using a 4-point Likert scale format ranging from ”strongly agree” to ”strongly disagree.” The RES had excellent internal consistency, with a Cronbach’s alpha of 0.82. The reliability coefficient for this study was 0.734.

#### 2.3.4. Satisfaction with Life Scale (SWLS)

This scale was developed by Diener et al. [20] to assess the cognitive components of subjective well-being. It includes a 5-item scale that evaluates an individual’s overall level of life satisfaction. Respondents answer using a 7-point scale that ranges from 7 (strongly agree) to 1 (strongly disagree). Items are summed to yield a total score for life satisfaction. The total scores ranged from 5 to 35, with higher scores indicating greater life satisfaction. Scores may be interpreted as follows: 5–9 (extremely dissatisfied), 10–14 (dissatisfied), 15–19 (slightly dissatisfied), 20 (neutral), 21–25 (slightly satisfied), 26–30 (satisfied), and 31–35 (extremely satisfied). In this study, the reliability coefficient was 0.902.

#### 2.3.5. Revised Death Anxiety Scale (RDAS)

The scale was developed by Thorson and Powell [27] and consisted of a 25-item questionnaire using both true–false and five-point Likert response formats. It was subdivided into four subscales, which consist of low death anxiety, afterlife concerns, helplessness and bodily integrity, and fear of pain. Higher scores indicate higher levels of death anxiety. The RDAS demonstrated excellent internal consistency in this study, with a Cronbach’s alpha of 0.958, consistent with a previous validation study [28].

#### 2.3.6. Beck Depression Inventory (BDI)

The BDI was a 21-item scale developed by Beck et al. [29]. It was a self-report rating questionnaire that assessed depression-related attitudes and symptoms. Each question had a set of at least four possible responses (0 to 4), ranging in intensity. The scale exhibits high internal consistency, with alpha coefficients of 0.86 and 0.81 for both psychiatric and non-psychiatric populations, respectively. This scale was divided into 2 subscales: the cognitive–affective subscale (Items 1–8) and the somatic-performance subscale (Items 9–21). In this study, the reliability coefficient was 0.888.

### 2.4. Data Collection Procedure

The researcher submitted a letter to the barangay officials requesting permission to conduct research and distribute questionnaires to older adults in the community to collect the necessary information. The researchers coordinated with the barangay captains and the office of the senior citizen association in the selected city to determine the optimal time to select participants based on the established inclusion and exclusion criteria. Given the COVID-19 pandemic restrictions, the researchers identified a liaison officer in the target area. The liaison officer conducted data gathering on behalf of the researchers. Before this step, the researchers provided guidance and instructions on how to perform the data gathering. For the instruments used in the study, the researchers emailed the original author requesting permission to use them. Participants were given time to complete the self-administered questionnaire. The researchers ensured the implementation of safety protocols, given that the respondents were older adults who were more vulnerable during the pandemic. They were required to stay at home and maintain a social distance from other people.

The researchers initially approached a total of 150 eligible older adults in coordination with barangay officials and the OSCA. Of these, 119 agreed to participate and completed the survey, yielding a response rate of 79.3%. Non-participation was primarily due to lack of interest, unavailability during data collection, or reluctance to complete the self-administered questionnaires. This recruitment process enhances transparency and allows for a clearer assessment of potential non-response bias [24]. The instruments were primarily self-administered. However, trained liaison personnel provided clarification when needed, ensuring consistent and complete responses.

### 2.5. Data Analysis

JAMOVI version 2.7.6 was used for data analysis. To summarize the study variables and participant demographics, descriptive statistics (frequencies, percentages, means, and standard deviations) were used. The bivariate correlations between meaning in life, self-esteem, life satisfaction, depression, and death anxiety were examined using Pearson’s product-moment correlation coefficients.

Multiple linear regression analyses were used to identify the independent predictors of depression and death anxiety. For each outcome variable, life satisfaction, self-esteem, and meaning in life were simultaneously incorporated as predictors in different models. The model fit was assessed using R^2^, adjusted R^2^, and the F statistic. Assumptions of normality were assessed using the Kolmogorov–Smirnov test (*p* > 0.05), indicating that the data were approximately distributed normally. Statistical significance was set at *p* < 0.05.

## 3. Results

Table 1 shows the participants’ demographic profiles. The mean age of the respondents was 68.32 (±7.25). The majority of participants in this study were female (67.2%) and married (50.4%). Most participants were Catholics (92.4%), had completed elementary school (44.5%), and reported low monthly income (60.5% earning less than PHP 9520). These characteristics suggest that the sample largely represents community-dwelling older adults with modest socioeconomic status.

As shown in Table 2, the overall mean score for meaning in life among participants was 5.83 (±0.97). Specifically, its subscales, which include presence and search, yielded mean scores of 6.03 (±0.86) and 5.71 (±1.30), respectively. In addition, the mean self-esteem rating was 2.14 (±0.37), while the subscales self-confidence and self-depreciation had mean scores of 2.49 (±0.42) and 1.78 (±0.50), respectively. The mean life satisfaction score was 6.18 (±0.78). On the other hand, the overall mean score of death anxiety was 3.50 (±0.52), while its subscale had a mean score of 3.78 (±0.74) for low death anxiety, 2.98 (±0.70) for afterlife concern, 3.31 (±0.60) for hopelessness and bodily integrity, and 3.86 (±0.60) for fear of pain load. Additionally, the mean score for depression was 0.55 (±0.389), while its subscales, cognitive–affective and somatic-performance, had mean scores of 0.45 (±0.38) and 0.61 (±0.43), respectively.

The relationship of meaning in life, self-esteem, and life satisfaction with death anxiety and depression was measured using Pearson’s r correlation, as shown in Table 3. Significant relationships were observed between death anxiety and meaning in life (r = 0.437, *p* < 0.001) and life satisfaction (r = 0.432, *p* < 0.001), indicating moderate positive associations. No significant relationship was found between self-esteem and death anxiety (r = 0.133, *p* = 0.151). In contrast, depression showed significant negative correlations with meaning in life (r = −0.516, *p* < 0.001), self-esteem (r = −0.328, *p* < 0.001), and life satisfaction (r = −0.569, *p* < 0.001), reflecting moderate inverse relationships.

As shown in Table 4, a multiple regression analysis was used to examine predictors of death anxiety and depression. For death anxiety, the overall model was statistically significant, F (3115) = 10.36, *p* < 0.001, explaining 21.3% of the variance (R^2^ = 0.213). Both meaning in life (B = 0.135, *p* = 0.025) and life satisfaction (B = 0.196, *p* = 0.017) emerged as significant positive predictors of death anxiety. This indicates that higher levels of meaning in life and life satisfaction are associated with increased death anxiety. In contrast, self-esteem was not a significant predictor (*p* = 0.083), suggesting that its effect does not independently contribute to death anxiety when other variables are controlled.

For depression, the regression model was also statistically significant, F (3115) = 20.73, *p* < 0.001, accounting for 35.1% of the variance (R^2^ = 0.351). Both life satisfaction (B = −0.268, *p* < 0.001) and meaning in life (B = −0.117, *p* = 0.005) were significant negative predictors of depression, indicating that higher levels of these variables are associated with lower depressive symptoms. Self-esteem was not a significant predictor of depression (*p* = 0.258), indicating that it does not independently predict depression when controlling for other variables.

## 4. Discussion

This study expanded on previous correlational findings by examining the associations between meaning in life, self-esteem, life satisfaction, death anxiety, and depression among older Filipino adults. The findings provide a more complex view of how these psychological resources specifically impact mental health outcomes in later life. Meaning in life and life satisfaction were found to be significantly associated with both death anxiety and depression. These results emphasize the intricate and situation-specific role of existential and evaluative well-being in aging.

The most significant independent predictor of depression was found to be life satisfaction, which aligns with a large body of research demonstrating a substantial correlation between emotional well-being in older adulthood and subjective assessments of life quality [20,23]. This finding is consistent with prior research demonstrating that higher life satisfaction is associated with lower depressive symptoms across diverse older adult populations, reflecting the protective role of cognitive well-being in emotional regulation [9,20]. Cumulative evaluations of social stability, family ties, and health, the domains that become more important as one ages, reflect life happiness. Longitudinal and cross-sectional research conducted across Asian populations revealed that higher life satisfaction is linked to lower depressive symptoms, even in older individuals with chronic illness or functional decline [6,30].

Meaning in life independently predicted lower depression, supporting existential and lifespan developmental theories that highlight purpose as a crucial psychological resource in aging [11,12]. Older adults with a strong sense of meaning are better equipped to make sense of age-related losses, thereby maintaining their resilience and hope. Higher meaning in life is consistently linked to lower depressive symptoms in both community-dwelling and institutionalized older adults, often through improved coping, increased acceptance, and a greater perceived value of life experiences [5,13].

However, despite its bivariate association, self-esteem was not a significant predictor of depression in the regression model. This result is consistent with meta-analytic and longitudinal research indicating that self-esteem may have an indirect impact on depression through broader constructs like life satisfaction, meaning in life, or social connectedness, rather than acting as an independent protective factor in later adulthood [31,32]. As people age, their sense of self-esteem may become less reliant on their accomplishments and more closely linked to their relationships and overall sense of life contentment.

A more complex pattern emerged from the regression analysis of death anxiety, with higher levels of death anxiety being independently predicted by life satisfaction and meaning in life. While previous research has found a negative association between meaning and death anxiety [5], new research indicates that people who believe their lives are fulfilling and meaningful may feel more anxious about dying when important aspects of their lives, such as family stability, autonomy, or health, are in jeopardy [24]. According to this viewpoint, fear of loss rather than existential emptiness may be the cause of death anxiety, which is consistent with theoretical claims that a high psychological investment in life might heighten mortality awareness [3].

These findings should be interpreted with caution, as the observed positive associations may be influenced by unmeasured factors, such as increased awareness of loss, health concerns, or heightened mortality salience. Methodological considerations, including shared method variance and the use of self-report measures, may also have contributed to these relationships [33].

The observed positive association between psychological resources and death anxiety within a cultural context can also be interpreted. In collectivist societies, the self is often defined in relation to family, spirituality, and social roles, which shape how individuals interpret aging and mortality [19]. In the Filipino context, where strong family obligations (utang na loob), religious practices, and intergenerational co-residence are common, death anxiety may reflect concerns about fulfilling familial roles, maintaining spiritual preparedness, and avoiding burdening family members [18].

Broader global and local contexts may also help us understand the present findings. Globally, mental health among older adults has been identified as a major public health priority, particularly as populations age and the burden of depression increases [7]. In the Philippines, older adults often rely on family-based support systems, which may shape both psychological resilience and vulnerability. At the community level, access to health services and opportunities for social engagement may further influence mental health outcomes in later life.

From a theoretical perspective, these findings support frameworks that view psychological well-being and mortality awareness as interconnected rather than opposing constructs. In practice, the results emphasize the necessity of interventions that address both the emotional and existential dimensions of care, including life review, meaning-centered approaches, and community-based psychosocial programs tailored to older adults.

The positive association between life satisfaction and death anxiety is also supported by studies showing that those who place a high value on life may fear death since it means being separated from important roles and relationships [4,34]. In this context, death anxiety may reflect concern over unfulfilled responsibilities rather than psychopathology, particularly among individuals who remain actively engaged in meaningful roles and relationships [18,28]. Taken together, these findings underscore the dual and context-dependent roles that life satisfaction and meaning in life play in later-life mental health. Rather than functioning solely as protective factors, these psychological resources may simultaneously enhance emotional well-being while heightening awareness of mortality. These results highlight the importance of interventions that support both psychological resilience and the management of existential concerns associated with aging. These findings align with global health priorities emphasizing healthy aging and mental well-being, as outlined by the World Health Organization [7]. Addressing mental health among older adults also contributes to Sustainable Development Goal 3, which promotes health and well-being across the lifespan.

## 5. Limitations

When interpreting the findings, several limitations of this study should be considered. First, the use of convenience sampling from a single urban locality in Metro Manila, along with a relatively small sample size (*n* = 119), limits the generalizability of the results and may affect the stability of the regression estimates. As such, the findings may not fully represent older adult populations in other regions of the Philippines, particularly those in rural areas with differing socioeconomic and healthcare conditions.

Second, the sample consisted only of community-dwelling older adults capable of completing self-administered questionnaires. Individuals with chronic illness or functional impairments or those in institutional care settings were not included. In addition, clinical variables such as comorbidities and disability status were not measured, despite their known associations with depression and death anxiety.

Third, the study relied on correlational and regression analyses, which, while appropriate for exploratory purposes, do not capture the complexity of relationships among variables. Interaction effects and demographic covariates were not included, and more integrative approaches, such as mediation analysis or structural equation modeling, may provide further insight into underlying mechanisms.

Fourth, all variables were assessed using self-report measures, which introduces the possibility of common-method bias that may inflate observed associations. In addition, although internal consistency reliability was established, further psychometric evaluation of the instruments in this population, such as factor structure, measurement invariance, and cultural adaptation, was not conducted.

Finally, the use of liaison personnel during data collection may have introduced variability in administration and potential response bias, despite efforts to standardize procedures.

Taken together, these limitations suggest that the findings should be interpreted with caution and may underestimate the psychological burden among more vulnerable subgroups of older adults. Future research should employ more diverse sampling strategies, include clinical and functional health indicators, and utilize more robust analytical approaches to better understand mental health in aging populations.

## 6. Conclusions

This study contributes to our knowledge of late-life mental health by demonstrating how important psychological resources operate in complex and culturally rooted ways among older Filipino individuals. Meaning in life and life satisfaction appear to simultaneously influence existential concerns and emotional well-being, reflecting the complex psychological landscape of aging rather than functioning as consistently protective variables. These results underscore the importance of considering the interplay between emotional, existential, and cultural factors to fully comprehend well-being in later life.

The findings emphasize the importance of acknowledging death anxiety as a possible manifestation of ongoing engagement with life, important relationships, and personal obligations, in addition to being a sign of distress. Such existential worries may be entwined with spiritual beliefs and relational responsibilities in collectivist societies, indicating that death anxiety may coexist with psychological well-being rather than immediately contradict it. This viewpoint necessitates a more careful interpretation of existential experiences in older populations.

In practice, interventions for older adults should go beyond symptom reduction to include methods that enhance existential dialogue, life review, and meaning making. By providing relationship-centered, culturally sensitive treatment that acknowledges both emotional resilience and mortality awareness, mental health and nursing providers are well-positioned to address these issues effectively. To better understand how psychological resources change throughout the aging process and to develop techniques that support well-being, dignity, and existential comfort in later life, future research employing longitudinal and intervention-based designs will be crucial.

## 7. Implications

The limited sample size and convenience sampling necessitate cautious interpretation of the findings, despite their usefulness as preliminary insights.

The results of this research indicate the importance of improving mental health services for older adults, especially in community settings where most Filipino older adults live. To ensure early diagnosis and support for at-risk seniors, local government units and barangay health centers should incorporate regular screening for depression and death anxiety into their senior citizens’ programs. Treatments for older adults should include interventions that foster purpose, spirituality, remembrance, and meaningful social involvement since meaning in life and life satisfaction have emerged as important psychological resources. Programs such as life review sessions, elder volunteer programs, and family-oriented spiritual or cultural activities may promote stronger psychological resilience.

Empowerment-focused care, opportunities for autonomy, and recognition of their contributions to society can strengthen the feelings of self-worth among senior citizens. However, the complex relationship between meaning, life satisfaction, and death fear emphasizes the necessity of culturally sensitive strategies to address the existential issues of older adults. Policies and initiatives must bolster social support networks, encourage intergenerational relationships, and guarantee accessible healthcare to reduce anxieties associated with deteriorating health or dependency, as Filipino senior citizens frequently derive meaning from family, spirituality, and independence. To enable frontline healthcare personnel to effectively meet the psychological and spiritual needs of older adults, training programs should incorporate courses on existential care, communication skills, and spiritual assessment. Lastly, to guide evidence-based policies targeted at enhancing the well-being and dignity of Filipino older adults, policymakers and researchers should fund long-term and intervention-based studies to further investigate how improving purpose, self-esteem, and satisfaction could mitigate depression and death anxiety.

## Figures and Tables

**Table 1:** Demographic Profile of the Respondents (*n* = 119).

Profile	Frequency (*f*)	Percentage (%)	Mean (SD)
Age (Years)			68.32 (±7.25)
Sex			
Male	39	32.8	
Female	80	67.2	
Civil Status			
Single	18	15.1	
Married	60	50.4	
Widowed	41	34.5	
Religion			
Catholic	110	92.4%	
Non-Catholic	9	7.6%	
Educational Attainment			
Elementary Graduate	53	44.5	
High school Graduate	46	38.7	
College Graduate	20	16.8	
Monthly Income			
Less than PHP 9520	72	60.5	
PHP 9520 to PHP 19,040	36	30.3	
PHP 19,040 and above	11	9.2	

**Table 2:** Meaning in Life, Self-Esteem, and Life Satisfaction; Death Anxiety and Depression among the participants (*n* = 119).

	Mean	Standard Deviation
Meaning in Life	**5.83**	**±0.97**
Presence	6.03	±0.86
Search	5.71	±1.30
Self-esteem	**2.14**	**±0.37**
Self-confidence	2.49	±0.42
Self-depreciation	1.78	±0.50
Life Satisfaction	**6.18**	**±0.78**
Death Anxiety	**3.50**	**±0.52**
Low in death anxiety	3.78	±0.74
Afterlife concern	2.98	±0.70
Helplessness and bodily integrity	3.31	±0.60
Fear of pain load	3.86	±0.60
Depression	**0.55**	**±0.389**
Cognitive–affective	0.45	±0.38
Somatic-performance	0.61	±0.43

Note: Bold values represent overall mean scores of the main study variables.

**Table 3:** Relationship of Meaning in Life, Self-esteem, and Life Satisfaction to Death Anxiety and Depression.

	Death Anxiety		Depression	
	r-Coefficient	*p*-Value	r-Coefficient	*p*-Value
Meaning in Life	0.437	* 0.000	−0.516	* 0.000
Self-esteem	0.133	0.151	−0.328	* 0.000
Life Satisfaction	0.432	* 0.000	−0.569	* 0.000

* *p*-value was significant at the 0.01 level.

**Table 4:** Multiple Linear Regression Models Predicting Depression and Death Anxiety Among Filipino Older Adults (*n* = 119).

Variable	B	SE	t	*p*
Death Anxiety Model				
Constant	2.014	0.371	5.43	<0.001
Meaning in Life	0.135	0.060	2.27	0.025
Self-esteem	−0.232	0.133	−1.75	0.083
Life Satisfaction	0.196	0.081	2.42	0.017
Model Fit: R^2^ = 0.213, Adj. R^2^ = 0.193, F (3115) = 10.36, *p* < 0.001				
Depression Model				
Constant				
Meaning in Life	−0.117	0.042	−2.83	0.005
Self-esteem	−0.102	0.090	−1.14	0.258
Life Satisfaction	−0.268	0.064	−4.21	<0.001
Model Fit: R^2^ = 0.351, Adj. R^2^ = 0.334, F (3115) = 20.73, *p* < 0.001				

## Data Availability

The data presented in this study are available upon request from the corresponding author. These data are not publicly available due to privacy restrictions.
